# On the Agreement between Manual and Automated Methods for Single-Trial Detection and Estimation of Features from Event-Related Potentials

**DOI:** 10.1371/journal.pone.0134127

**Published:** 2015-08-10

**Authors:** José A. Biurrun Manresa, Federico G. Arguissain, David E. Medina Redondo, Carsten D. Mørch, Ole K. Andersen

**Affiliations:** 1 Center for Sensory-Motor Interaction, Dept. of Health Science and Technology, Aalborg University, Aalborg, Denmark; 2 Departamento de Informática, Universidad Nacional de Entre Ríos, Oro Verde, Entre Ríos, Argentina; Lancaster University, UNITED KINGDOM

## Abstract

The agreement between humans and algorithms on whether an event-related potential (ERP) is present or not and the level of variation in the estimated values of its relevant features are largely unknown. Thus, the aim of this study was to determine the categorical and quantitative agreement between manual and automated methods for single-trial detection and estimation of ERP features. To this end, ERPs were elicited in sixteen healthy volunteers using electrical stimulation at graded intensities below and above the nociceptive withdrawal reflex threshold. Presence/absence of an ERP peak (categorical outcome) and its amplitude and latency (quantitative outcome) in each single-trial were evaluated independently by two human observers and two automated algorithms taken from existing literature. Categorical agreement was assessed using percentage positive and negative agreement and Cohen’s κ, whereas quantitative agreement was evaluated using Bland-Altman analysis and the coefficient of variation. Typical values for the categorical agreement between manual and automated methods were derived, as well as reference values for the average and maximum differences that can be expected if one method is used instead of the others. Results showed that the human observers presented the highest categorical and quantitative agreement, and there were significantly large differences between detection and estimation of quantitative features among methods. In conclusion, substantial care should be taken in the selection of the detection/estimation approach, since factors like stimulation intensity and expected number of trials with/without response can play a significant role in the outcome of a study.

## Introduction

Event-related potentials (ERPs) are synchronous voltage deflections in the EEG in response to external stimuli that reflect reception and processing of sensory information [1]. ERPs present excellent temporal resolution, in the order of milliseconds, providing an accurate estimation of the timing of processing activity in the brain. For many experimental applications and particularly in clinical settings, ERPs are commonly characterized by their polarity (positive or negative) and maximum voltage excursion (i.e., the peak amplitude), the time from stimulus onset to peak deflection (i.e., the peak latency) and the location of voltage changes across the head (i.e., the scalp distribution). Despite their simplicity, these features reflect surprisingly well the salient aspects of cerebral processing, and even more complex analyses can also be performed to gain insight into neurophysiological processes [2].

ERP amplitudes are a fraction of the magnitude of the background EEG, thus requiring further signal processing in order to enhance the signal-to-noise ratio. This is often performed by repeating the event of interest a number of times (from tens to thousands of trials, depending on the type of stimulus) and averaging the responses over time [3]. However, besides the obvious disadvantages associated with a large number of event repetitions, across-trial averaging may in some cases lead to distortion, inaccurate estimation or even loss of information of the ERP features [4]. The main reason for this is that not all relevant information is precisely time-locked to the event, leading to a certain level of variability in amplitudes and latencies, which might actually reflect fluctuations in signal transduction, expectation, attention or other cognitive processes [5].

In this regard, there is great interest in the development of single-trial methods for automated detection and estimation of ERP features, using a variety of different signal processing methods, including (but not limited to) wavelet denoising [6,7], independent component analysis [8,9], multiple linear regression [10], or combinations of these and other techniques [11]. Regardless of the approach, these methods are, in one way or another, validated against knowledge from human experts [12,13]. From here, an interesting question can be raised: how are the results and conclusions of a particular study affected if one method for ERP feature detection or estimation is used instead of another? Although a few attempts to address this issue have been performed [10,12], two questions remain largely unexplored: the agreement between humans and algorithms on whether an ERP is present or not after a stimulation (categorical agreement), and in the trials in which the ERP is indeed present, what is the variation in the estimated values of the relevant features (quantitative agreement).

The aim of this study was to establish reference values for the categorical and quantitative agreement between manual and automated methods for detection and estimation of ERP features. In particular, the study compares the performance of two experienced human observers and two existing and readily available algorithms for ERP feature detection. A detailed description of these methodologies will be presented in this paper, followed by a thorough comparison of their performances on the detection and estimation tasks. Finally, potential sources of disagreement and suggestions for improving the methods will be discussed in an effort to explain the main findings of the study.

## Materials and Methods

### Participants

Sixteen healthy male volunteers (23.6 ± 4.6 years) participated in a single experimental session. Written informed consent was obtained from all subjects, and the Declaration of Helsinki was respected. The study was approved by the local ethics committee of Region Nordjylland, approval number VN– 20110027.

### EEG recording

Continuous EEG data was recorded by a 128-channel system, using a standard EEG cap (Waveguard cap system, ANT-Software A/S, Enschede, Netherlands) based on the extended International 10–20 system. The common ground electrode was located along the sagital midline, between the Fz and FCz electrodes. The reference was set as the average of all unipolar electrodes. EEG data was filtered with a notch filter (50 Hz), sampled at 2048 Hz per channel and stored for offline analysis using ASA 4.7.3 (ANT-Software A/S, Enschede, Netherlands).

### Electrical stimulation

Electrical stimulation was performed through surface electrodes in order to evoke the ERPs [14]. The cathode (15 × 15 mm, type Neuroline 700, Ambu A/S, Denmark) was placed in the arch of the left foot, whereas the anode (50 × 90 mm, type Synapse, Ambu A/S, Denmark) was placed at the dorsum of the foot. Each stimulus consisted of a train of five constant-current pulses 1 ms pulse width, delivered at 200 Hz by a computer-controlled electrical stimulator (Noxitest IES 230, Aalborg, Denmark), that were perceived by the subjects as a single pricking stimulus. The stimulation was repeated with a random inter-stimulus interval ranging from 14 to 16 s. Stimulation intensities were normalized to the nociceptive withdrawal reflex threshold (RTh), i.e., the minimum current intensity required to elicit a withdrawal reflex from the tibialis anterior muscle, in order to titrate the stimulus intensity to an objective electrophysiological response. Six stimulation intensities were used: 0.50, 0.75, 1.00, 1.25, 1.50 and 2.00 times the RTh, to cover the full range of stimulation intensities commonly used in somatosensory assessment through electrical stimulation. This provided a full range of ERP response sizes, including some cases in which no response was elicited.

### Experimental procedure

Volunteers were comfortably placed in supine position with back support inclined 120° relative to the horizontal level. A pillow was placed under the knee to obtain approximately 30° knee joint flexion. Once the stimulation and recording electrodes were mounted, volunteers were thoroughly familiarized with electrical stimulation before any data was recorded. Afterwards, the RTh was obtained using a staircase procedure [15], and the stimulation intensities were derived. Five blocks containing 24 stimuli each (6 intensities x 4 repetitions per intensity) were applied with a 5 min interval between blocks (120 stimuli per subject in total). Each intensity level was applied 4 times within the same stimulation block (resulting in 20 stimuli per intensity) and all 24 stimuli within a block were presented in random order. Subjects were recurrently asked to keep their attention on the stimulus during the stimulation blocks.

### Data analysis

#### EEG signal processing

EEGLAB was used for offline EEG processing [16]. EEG data were filtered (band-pass 0.5–30 Hz), re-referenced to the linked mastoids M1 and M2, and divided into epochs of 2000 ms (200 ms pre-stimulus and 1800 ms post- stimulus). The mean amplitude of the pre-stimulus interval was used for baseline correction. Trials containing large artifacts were rejected after visual inspection. EEG epochs were further pre-processed using Independent Component Analysis (ICA) [17]. The resulting independent components (ICs) were visually inspected and those ICs that showed artifacts related to muscle activity or eye movements were eliminated [18]. EEG data was reconstructed from the remaining ICs, and single-trial EEG traces from the vertex (Cz) were subsequently used for automated and manual feature extraction.

#### Feature extraction

ERPs elicited by stimulation in the lower limb usually display three characteristic peaks [19,20]: a first negative peak (N1) at approximately 90 ms, followed by second negative deflection (N2) around 140 ms and a complex of positive waves, with a peak at approximately 250 ms (P2). Consequently, six features per trial were extracted from the ERPs, namely N1, N2 and P2 amplitudes and latencies. In order to extract these features, two different strategies were proposed: (1) a manual approach consisting on visual inspection and detection made by two experienced human observers and (2) an automated approach consisting of two automated single-trial detection algorithms. The results of the detection and estimation tasks can be found in S1 File.

In the manual strategy, two experienced blind observers (OBS1 and OBS2) carried out the manual detection of single-trial peaks. The observers worked at the same research institute and were trained in a similar way. They performed the manual detection using a custom-made program in MATLAB. The program displayed on a computer screen the mean ERP waveform across all intensities for the individual subject, from which the peak amplitudes and latencies were estimated manually by the observer based on their polarity and latency, using the built-in *Data Cursor* tool that MATLAB provides. Then, each single-trial waveform was visually inspected and its N1, N2 and P2 peaks were manually detected in the same fashion. The detected values were automatically stored by the program for further analysis. In order to avoid bias, the stimulation intensity in each particular trial was unknown to the observers, and manual detection was performed by the observers before any of the automated detection algorithms were evaluated.

In the automated strategy, two different methods were used for automated peak estimation: an algorithm based on the derivative of the signal that classifies using fuzzy logic (DRIV) inspired on previous work on automated detection of features in auditory evoked potentials [21], and an algorithm based on wavelet filtering and multiple linear regression (WVLT) [11]. The implementation of the DRIV algorithm was carried out in C++ (see description below) which is freely available at https://sourceforge.net/projects/stfderp, whereas the MATLAB implementation of the WVLT algorithm is freely available at http://iannettilab.webnode.com. There are several other examples of detection/estimation methods on the literature, many of which are more advanced that the two methods presented here [22–24]. However, these two methods were selected because they are readily available and represent two very different approaches to detection/estimation: the DRIV algorithm mimics the decision process performed by a human observer during a visual detection task, whereas the WVLT algorithm relies on a linear model of the filtered signal in order to estimate the amplitudes and latencies of the peaks at single-trial level.

*DRIV algorithm*: the first derivative of the signal was calculated using numerical differentiation, in order to detect all local maxima (for P2) and minima (for N1 and N2) in the ERP waveform. Maxima and minima were located in the points where the derivative of the signal changes its sign (from positive to negative or vice versa). All the local maxima/minima found in each trial were further weighted within three fuzzy zones defined for the N1, N2 and P2 peaks. Each fuzzy zone had a central latency and two boundaries. The fuzzy weights were defined by two quadratic functions that depended on the corresponding peak latency found in the average ERP (central latency) and its expected variability (boundaries), as reported in previous articles [19,25,26]. Consequently, the fuzzy zones had a maximum weight at the central latency that decreased in a quadratic fashion towards the boundaries, and the weight was set to zero outside the boundaries. The weighting process was performed by multiplying the ERP amplitude of the maxima/minima with the weight given by the correspondent fuzzy zone. The resulting maximal values for each zone were then selected as peaks, under the condition that their amplitude values were negative (for N1 and N2) or positive (for P2). If the same minimum value was a candidate for N1 and N2 simultaneously, the decision was performed based on which of the central latencies of the corresponding fuzzy windows was closest to this value. Once this value was assigned to N1 or N2, the remaining minimum values within the fuzzy window of the other peak were subsequently analysed in order to find the remaining peak; if there were no more candidates, the remaining peak was categorized as absent.

*WVLT algorithm*: initially, the single-trial ERP signals were represented in the time-frequency domain using the continuous Morlet wavelet transform (CWT, bandwidth parameters f_b_ = 0.05 and f_0_ = 6) and squared to obtain the magnitude of their power spectrum. These representations were then averaged, resulting in a time-frequency matrix that was further thresholded to obtain a binary mask. This mask was applied to each single-trial time–frequency representations to filter out wavelet coefficients with low energy. The filtered single-trial ERPs in the time domain were then reconstructed by using the inverse continuous wavelet transform (ICWT). The automated detection of N1, N2 and P2 amplitudes and latencies was performed using a multiple linear regression approach [10]. Two regressors per peak (signals in the time domain and their first derivative) were obtained for each subject from the filtered average ERP. In order to obtain the regressors for each peak, the average ERP was separated where the voltage signal equalled zero. Since ERPs measured at the vertex usually present an overlap between N1 and N2 peaks without a zero-crossing point between them, the procedure was performed twice for each subject, selecting only one negative peak each time (N1 or N2) together with a single positive peak (P2 both times). The selection was made by determining manually the latency of the aforementioned peaks in the average ERP. Each single-trial ERP is then fitted with the set of regressors obtained from the averaged ERP using a least squares approach. The single-trial amplitudes and latencies of the N1, N2 and P2 peaks were then obtained from the fitted regressors by measuring the maximum voltage peaks within a time window centred on the latency of each average ERP peak previously determined.

#### Agreement

*Categorical data (presence/absence of a peak)*: the categorical agreement for all possible pairings of manual and automated methods was assessed using overall percent agreement (*p*_*o*_), positive and negative percent agreement (*p*_*pos*_ and *p*_*neg*_, respectively), chance percent agreement (*p*_*e*_) and Cohen’s kappa (κ). In relation to these indexes, *p*_*o*_ represents the sum of all trials in which the methods agree divided by the total number of trials, whereas *p*_*pos*_ is calculated from the number of positive trials (i.e. a peak is present) in which both methods agree on divided by all of the positive trials for both methods and *p*_*neg*_ is calculated from the number of negative trials (i.e. a peak is absent) in which both methods agree on divided by all of the negative trials for both methods. The last two indices provide information about the type of decision (i.e. presence or absence of a peak) on which the methods disagree more. Finally, *p*_*e*_ is calculated as the sum of the joint positive and negative responses, and represents the level of agreement that would still be present if the methods decided randomly on the presence/absence of a peak. From that definitions, κ is calculated as the ratio between the overall percent agreement corrected for chance (*p*_*o*_—*p*_*e*_), divided by the maximum possible percent agreement corrected for chance (100%–*p*_*e*_). Normally, κ ranges from 0 (no agreement beyond chance) to 1 (perfect agreement), although it is possible to observe negative values of κ (when the agreement between methods is worse than what would be expected by random decisions). In these cases, the lower limit of κ was set to zero. Cohen’s κ, together with *p*_*pos*_ and *p*_*neg*_ were selected as the primary outcomes for categorical agreement, in line with current recommendations [27]. The remaining indexes (*p*_*o*_ and *p*_*e*_) are reported as secondary outcomes to provide reference and context, in order to better understand how Cohen’s κ is derived and what would happen if only *p*_*o*_ was quantified without taking *p*_*e*_ into account.

*Quantitative data (variation in peak amplitudes/latencies)*: the absolute variation of peak features (amplitudes/latencies) between all possible pairings of manual and automated methods was assessed using Bland-Altman analysis. This method considers the differences between features (amplitudes/latencies) estimated by two methods from the same single-trial ERP recording. The mean difference is called bias (an index of *systematic error*), and the standard deviation of these differences provides a reference of the absolute variation between methods (an index of *random error*). Approximately 95% of the differences should lie between ± 1.96 standard deviations, which are regarded as the limits of agreement (LoA) [28]. The LoA provide reference values of the maximum differences that can be expected between methods when measuring the same quantity. For the sake of clarity, only the absolute values of the bias and the LoA were reported (the analysis focused on how large the differences between methods were and not on the specific sign of these differences). Additionally, the intraclass correlation coefficient (ICC) and the coefficient of variation (CV) were calculated. The ICC reflects the proportion of variance of an estimation due to variability between trials [29]. For this analysis, a two-way mixed model using absolute agreement was selected, and the ICC of single measurements was reported. Furthermore, the CV represents the variability within trials as a percentage of the average estimation [30]. Bland-Altman LoA and CV were selected as the primary outcomes to assess quantitative agreement, in line with current recommendations [31]. The remaining index (ICC) is reported as secondary outcome for reference/comparison purposes, since a large number of studies use it as main variable for decision-making.

#### Statistics

*Hypotheses and data arrangement*: the main hypothesis was that there exist differences on the categorical and quantitative agreement between different pairings of human observers and automatic methods for ERP feature detection and estimation. In order to test this hypothesis, all indexes (*p*_*o*_, *p*_*e*_, *p*_*pos*_, *p*_*neg*_, Cohen’s κ, bias and LoA, CV and ICC) were derived for all possible pairings between manual and automated methods (OBS1-DRIV, OBS1-WVLT, DRIV-WVLT, OBS2-DRIV, OBS2-WVLT, OBS1-OBS2). Each index was calculated using all available trials from each subject (approx. 120 trials, without distinction between stimulation intensities), resulting in samples of sixteen index values (*n* = 16) for each possible pairing for each feature (N1, N2 and P2). A secondary hypothesis was that the categorical and quantitative agreement of different pairings of human observers and automatic methods for ERP feature detection and estimation are affected by stimulation intensity. In order to test this hypothesis, two representative indexes for categorical and quantitative agreement (Cohen’s κ and CV, respectively) were subsequently derived from the best- and worst-performing pairings between manual and automated methods taking into account the stimulation intensity as a factor (approx. 20 trials per intensity per subject), resulting in samples of sixteen index values (*n* = 16) for each possible stimulation intensity within a specific pairing (best- or worst-performing) for each feature (N1, N2 and P2). Cohen’s κ was selected as the preferred index for categorical agreement because it is a single summary measure (unlike *p*_*pos*_ and *p*_*neg*_ that have to be analyzed together) that takes into account agreement by chance. In time, CV was selected as the preferred index for quantitative agreement because it is also a single summary measure (unlike bias and LoA) and it is normalized as a percentage, which is desirable since absolute differences are expected to be larger at higher stimulation intensities (since the ERP responses themselves are also larger).

*Tests*: statistical analysis was performed using SigmaPlot 11 (Systat Software, Inc., USA). In general, the distributions of the indexes were not normally distributed, so differences in categorical and quantitative agreement between pairings and differences in categorical and quantitative agreement due to stimulation intensity within a specific pairing were assessed using Friedman’s test; when a significant difference was found, post hoc pairwise comparisons were carried out using Student-Newman-Keuls (SNK) test. P values smaller than 0.05 were regarded as significant.

## Results

### Descriptive statistics

**Fig 1:**
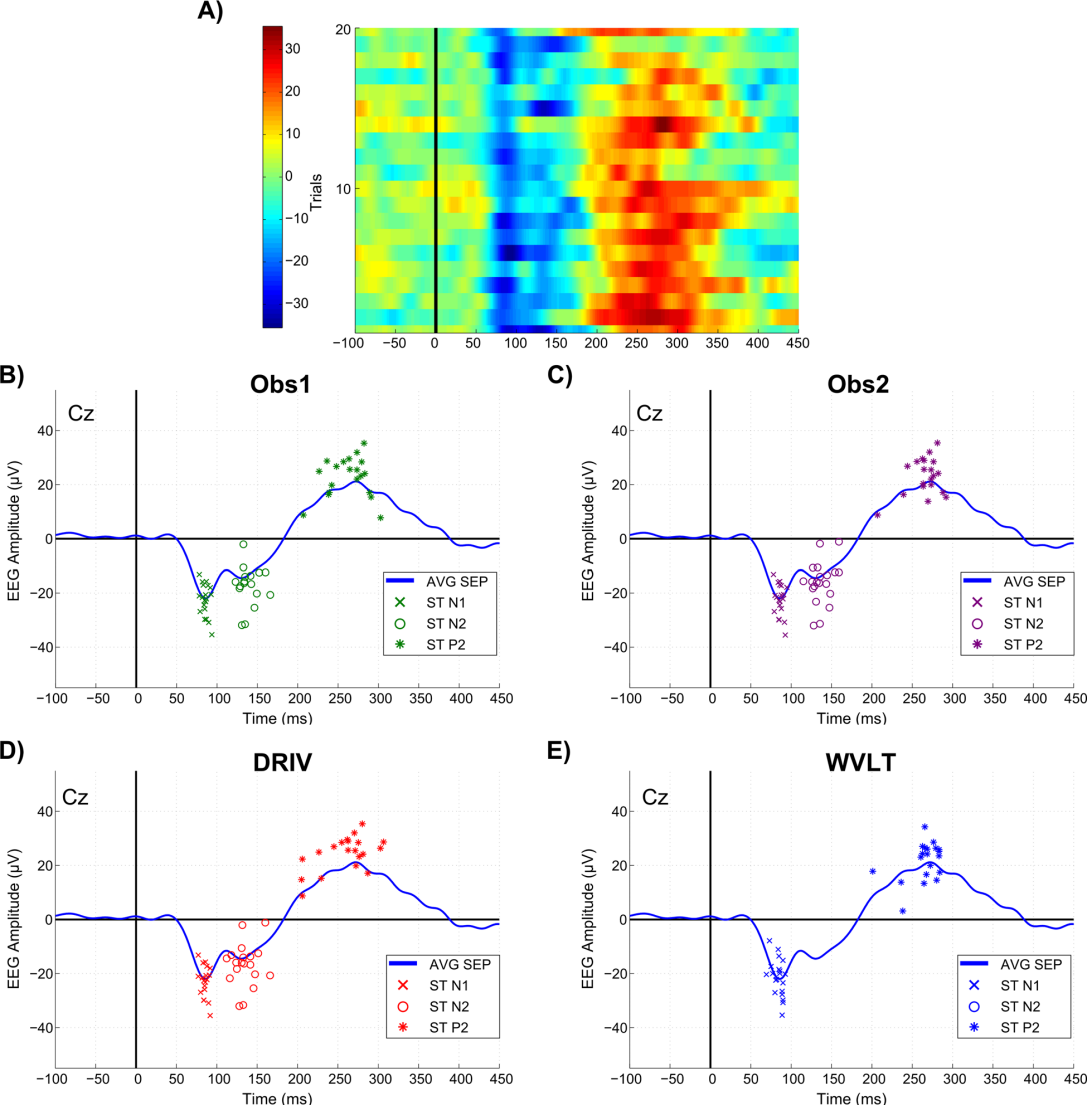
Comparison of manual and automated ERP feature detection/estimation methods. **A)** Trial-by-trial image of ERP responses of a single subject elicited with the highest stimulation intensity (20 trials). **B-E)** Performance of OBS1, OBS2, DRIV and WVLT methods, respectively, on the detection/estimation of single-trial ERP features of a single subject elicited with the highest stimulation intensity. Crosses, circles and asterisks represent single-trial N1, N2 and P2 features, respectively, while the blue trace is the average of 20 trials. Note that WVLT algorithm did not detect the N2 peak in **E)**.

The average RTh was 8.3 ± 3.8 mA. After pre-processing, a total of 1896 trials were subsequently analyzed (24 trials eliminated in total), averaging 118.5 ± 1.6 trials per subject. Table 1 shows the average single-trial peak amplitude and latency values and the number of peaks detected with each method, whereas Fig 1 presents a comparison of the performance of manual and automated ERP feature detection/estimation methods for a typical subject.

**Table 1:** Single-trial peak amplitude and latency values and number of peaks detected with each method, averaged across stimulation intensities and subjects.

	OBS 1	OBS 2	DRIV	WVLT
*N1 peak*				
Amplitude (μV)	-18.5 ± 7.4	-17.9 ± 7.2	-19.3 ± 6.9	-13.8 ± 9.1
Latency (ms)	93.0 ± 20.8	94.0 ± 22.6	101.2 ± 21.2	88.8 ± 20.9
Number of peaks detected	96.4 ± 15.1	97.8 ± 15.9	96.6 ±22.0	93.8 ± 47.4
*N2 peak*				
Amplitude (μV)	-19.5 ± 4.6	-18.3 ± 4.1	-18.3 ± 5.2	-14.4 ± 10.3
Latency (ms)	146.0 ± 13.6	143.1 ± 12.4	147.6 ± 12.3	137.2 ± 33.4
Number of peaks detected	64.5 ± 45.6	69.0 ± 44.9	70.9 ± 43.7	48.6 ± 55.6
*P2 peak*				
Amplitude (μV)	24.0 ± 6.4	24.6 ± 7.4	25.5 ± 6.1	20.6 ± 5.7
Latency (ms)	254.0 ± 16.2	255.4 ± 17.5	262.3 ± 11.9	265.2 ± 23.7
Number of peaks detected	109.4 ± 10.0	104.4 ± 13.9	116.2 ± 3.1	118.5 ± 1.6

Values are presented as mean ± SD.

### Categorical agreement

#### N1 peak

**Fig 2:**
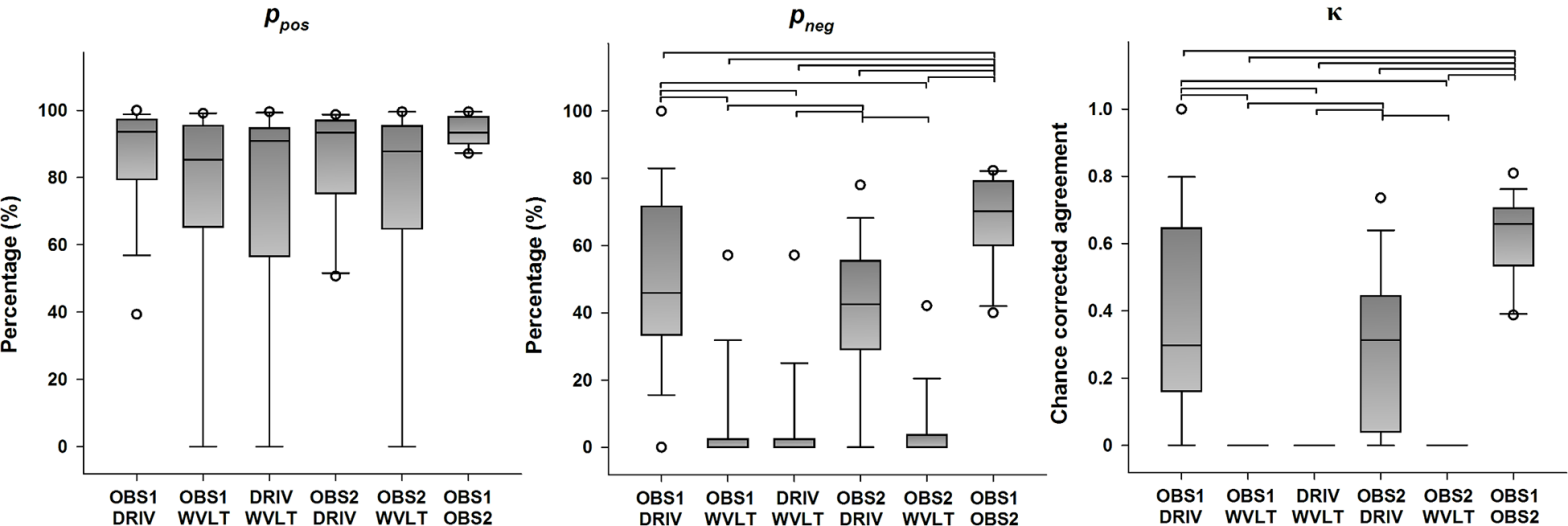
Categorical agreement in the detection of the N1 peak (primary outcomes). The boundaries of the boxes indicate the 25^th^ and 75^th^ percentiles, the line within the box marks the median, the whiskers indicate the 10^th^ and 90^th^ percentiles and the circles above and below represent outliers (*n* = 16 for each index). Horizontal lines on top of the bars represent statistically significant post hoc differences between pairings (Student-Newman-Keuls, *p* < 0.05). *p*_*pos*_: positive percent agreement, *p*_*neg*_: negative percent agreement, κ: Cohen’s kappa.

Descriptive statistics for the categorical agreement variables derived from all possible pairings of detection methods of the N1 peak are presented in Fig 2 (primary outcomes) and S1 Fig (secondary outcomes). Statistically significant differences in the categorical agreement between pairings were found for *p*_*o*_ (χ^2^(5) = 16.976, *p* = 0.005), *p*_*neg*_ (χ^2^(5) = 58.635, *p* < 0.001), *p*_*e*_ (χ^2^(5) = 16.436, *p* = 0.006) and κ (χ^2^(5) = 65.100, *p* < 0.001), whereas no significant differences were found for *p*_*pos*_ (χ^2^(5) = 10.838, *p* = 0.055). Post hoc analysis revealed that agreement between the human observers yielded significantly higher *p*_*o*_, *p*_*neg*_ and κ values compared to any other pairing (all *p* < 0.05). Additionally, all pairings between both human observers and the DRIV algorithm yielded significantly higher *p*_*neg*_ and κ values compared to all pairings between both human observers and the WVLT algorithm (all *p* < 0.05). Even though the Friedman test found a significant difference between the median *p*_*e*_ values between pairings, no significant differences were found in the post hoc tests (all *p* > 0.05).

#### N2 peak

**Fig 3:**
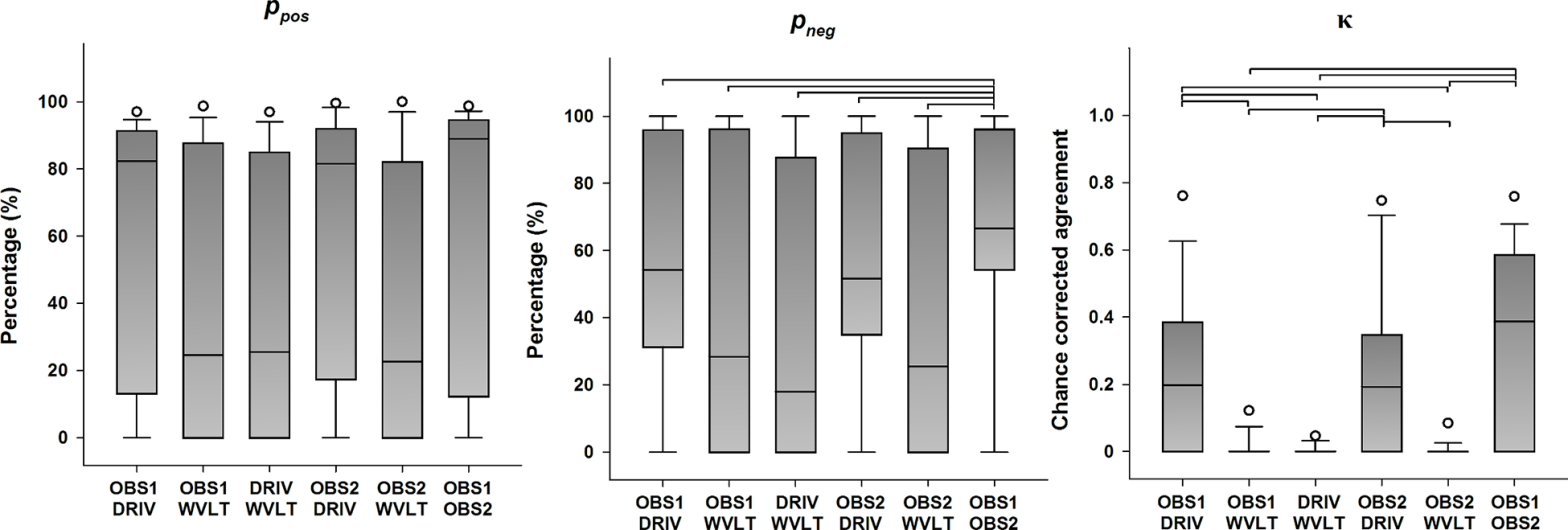
Categorical agreement in the detection of the N2 peak (primary outcomes). The boundaries of the boxes indicate the 25^th^ and 75^th^ percentiles, the line within the box marks the median, the whiskers indicate the 10^th^ and 90^th^ percentiles and the circles above and below represent outliers (*n* = 16 for each index). Horizontal lines on top of the bars represent statistically significant post hoc differences between pairings (Student-Newman-Keuls, *p* < 0.05). *p*_*pos*_: positive percent agreement, *p*_*neg*_: negative percent agreement, κ: Cohen’s kappa.

Descriptive statistics for the categorical agreement variables derived from all possible pairings of detection methods of the N2 peak are presented in Fig 3 (primary outcomes) and S2 Fig (secondary outcomes). Statistically significant differences in the categorical agreement between pairings were found for *p*_*o*_ (χ^2^(5) = 15.695, *p* = 0.008), *p*_*pos*_ (χ^2^(5) = 13.719, *p* = 0.017), *p*_*neg*_ (χ^2^(5) = 24.169, *p* < 0.001), and κ (χ^2^(5) = 43.459, *p* < 0.001), whereas no significant differences were found for *p*_*e*_ (χ^2^(5) = 3.230, *p* = 0.665). Post hoc analysis revealed that agreement between the human observers yielded significantly higher *p*_*neg*_ values compared to any other parings (all *p* < 0.05). Furthermore, all possible pairings between the human observers and the DRIV algorithm yielded significantly higher κ values than any pairing involving the WVLT algorithm (all *p* < 0.05). Even though the Friedman test found a significant difference between the median *p*_*o*_ and *p*_*pos*_ values between pairings, no significant differences were found in any of the post hoc tests (all *p* > 0.05).

#### P2 peak

**Fig 4:**
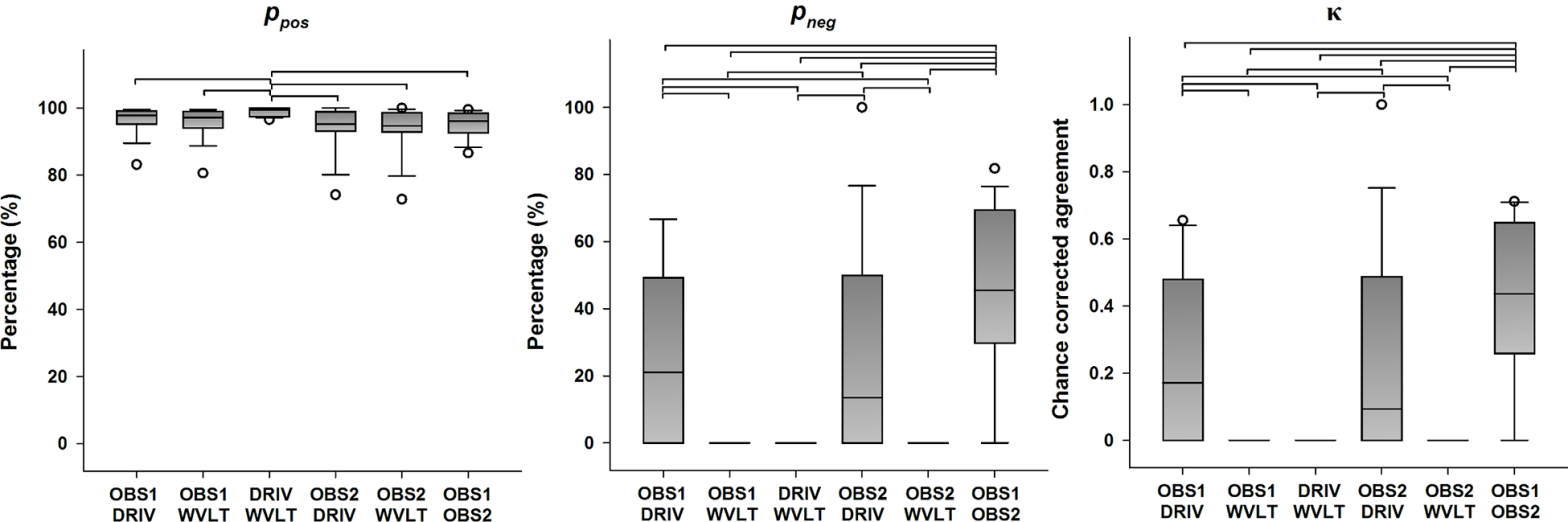
Categorical agreement in the detection of the P2 peak (primary outcomes). The boundaries of the boxes indicate the 25^th^ and 75^th^ percentiles, the line within the box marks the median, the whiskers indicate the 10^th^ and 90^th^ percentiles and the circles above and below represent outliers (*n* = 16 for each index). Horizontal lines on top of the bars represent statistically significant post hoc differences between pairings (Student-Newman-Keuls, *p* < 0.05). *p*_*pos*_: positive percent agreement, *p*_*neg*_: negative percent agreement, κ: Cohen’s kappa.

Descriptive statistics for the categorical agreement variables derived from all possible pairings of detection methods of the P2 peak are presented in Fig 4 (primary outcomes) and S3 Fig (secondary outcomes). Statistically significant differences in the categorical agreement between pairings were found for *p*_*o*_ (χ^2^(5) = 32.985, *p <* 0.001), *p*_*pos*_ (χ^2^(5) = 3.340, *p <* 0.001), *p*_*neg*_ (χ^2^(5) = 49.444, *p* < 0.001), *p*_*e*_ (χ^2^(5) = 58.340, *p <* 0.001) and κ (χ^2^(5) = 49.276, *p* < 0.001). Post hoc analysis revealed that agreement between the two algorithms yielded significantly higher *p*_*o*_ and *p*_*pos*_ values compared to any other parings (all *p* < 0.05), but also significantly higher *p*_*e*_ values compared to any other parings (all *p* < 0.05). Additionally, all possible pairings between a human observer and any of the algorithms yielded significantly higher *p*_*e*_ values than the pairing of both human observers (all *p* < 0.05). Furthermore, the pairing between the human observers yielded significantly higher *p*_*neg*_ and κ values compared to all other possible pairings (all *p* < 0.05), and the pairings between either human observer and the DRIV algorithm resulted in significantly higher *p*_*neg*_ values compared to the pairings between either human observer and the WVLT algorithm (all *p* < 0.05).

### Quantitative agreement

#### N1 peak

**Fig 5:**
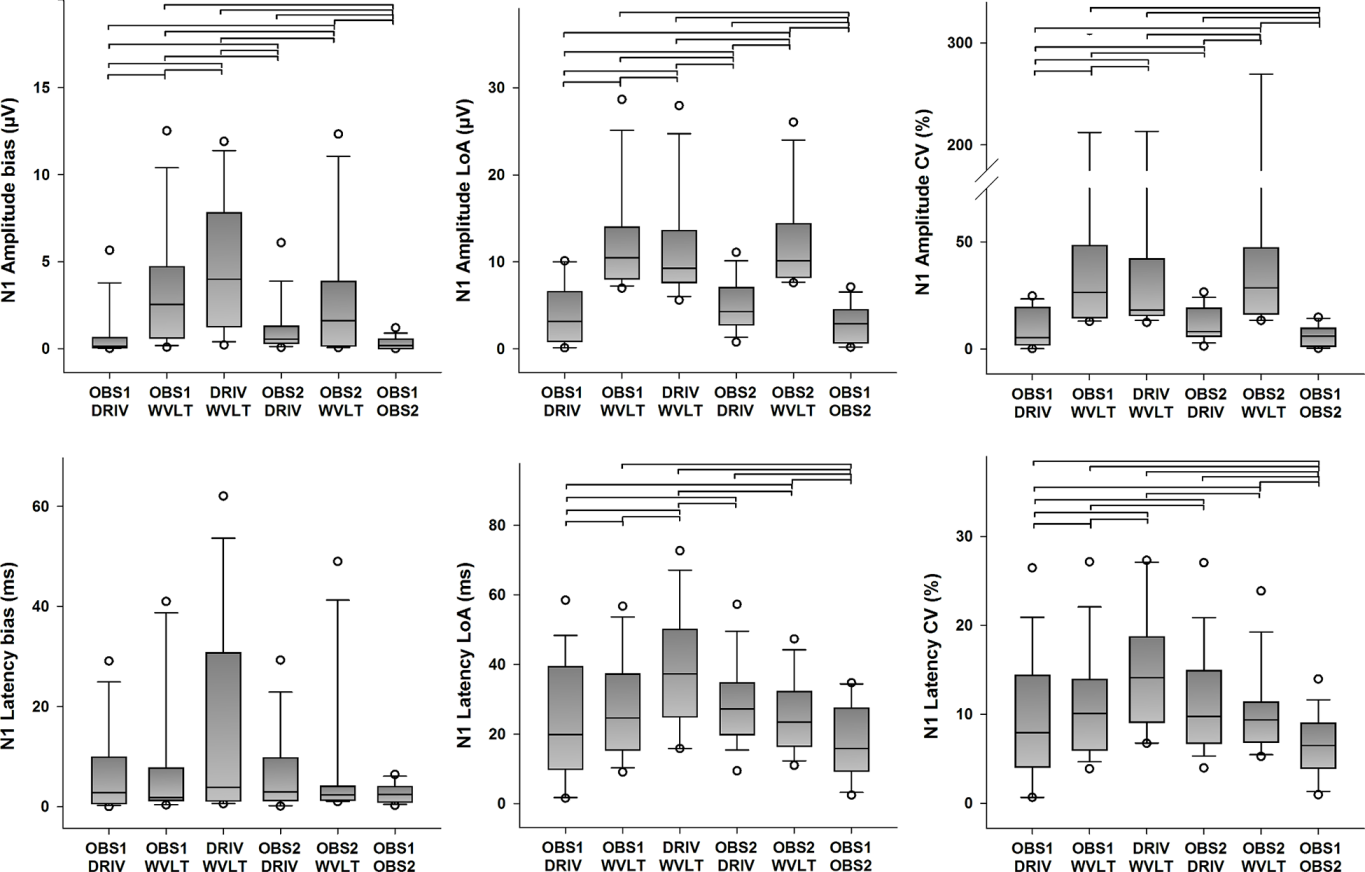
Quantitative agreement in the detection of the N1 peak (primary outcomes). The boundaries of the boxes indicate the 25^th^ and 75^th^ percentiles, the line within the box marks the median, the whiskers indicate the 10^th^ and 90^th^ percentiles and the circles above and below represent outliers (*n* = 16 for each index). Horizontal lines on top of the bars represent statistically significant post hoc differences between pairings (Student-Newman-Keuls, *p* < 0.05). LoA: limits of agreement, CV: coefficient of variation.

Descriptive statistics for the quantitative agreement from all possible pairings of detection methods of N1 amplitudes and latencies are presented in Fig 5 (primary outcomes) and S4 Fig (secondary outcome). Statistically significant differences in bias between pairings were found for N1 amplitudes (χ^2^(5) = 43.857, *p <* 0.001), but not for N1 latencies (χ^2^(5) = 5.000, *p* = 0.416). Moreover, statistically significant differences in the LoA between pairings were found for N1 amplitudes (χ^2^(5) = 41.000, *p <* 0.001), and for N1 latencies as well (χ^2^(5) = 25.791, *p <* 0.001). Post hoc analysis revealed several statistically significant differences in bias and LoA of N1 amplitude and latency estimations between pairings (see Fig 5 for details), but the error was consistently smaller for pairings between the human observers and the DRIV algorithm. Furthermore, statistically significant differences in ICC between pairings were found for N1 amplitudes (χ^2^(5) = 42.846, *p <* 0.001) and N1 latencies (χ^2^(5) = 26.595, *p* < 0.001). Likewise, statistically significant differences in CV between pairings were found for N1 amplitudes (χ^2^(5) = 49.967, *p <* 0.001), and for N1 latencies as well (χ^2^(5) = 29.308, *p <* 0.001). Post hoc analysis revealed several statistically significant differences in ICC and CV of N1 amplitude and latency estimations between pairings (see Fig 5 and S4 Fig for details), but in general the pairings between the human observers resulted in higher ICC and smaller CV values compared to the other possible pairings, followed by pairing between a human observer and the DRIV algorithm. Finally, pairings with the WVLT algorithm usually resulted in in lower ICC and higher CV values.

#### N2 peak

**Fig 6:**
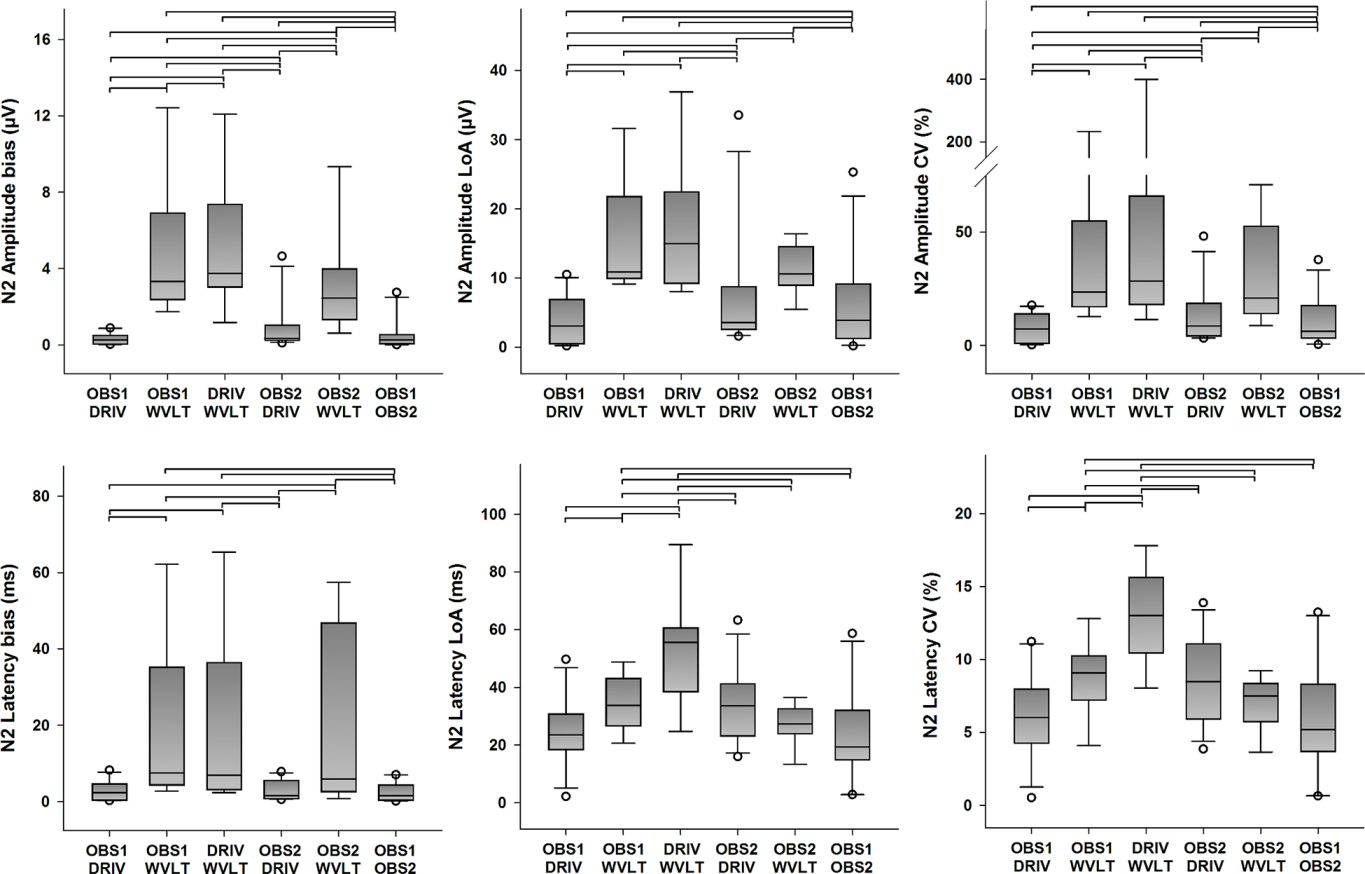
Quantitative agreement in the detection of the N2 peak (primary outcomes). The boundaries of the boxes indicate the 25th and 75th percentiles, the line within the box marks the median, the whiskers indicate the 10th and 90th percentiles and the circles above and below represent outliers (n = 16 for each index). Horizontal lines on top of the bars represent statistically significant post hoc differences between pairings (Student-Newman-Keuls, p < 0.05). LoA: limits of agreement, CV: coefficient of variation.

Descriptive statistics for the quantitative agreement from all possible pairings of detection methods of N2 amplitudes and latencies are presented in Fig 6 (primary outcomes) and S4 Fig (secondary outcome). Statistically significant differences in bias between pairings were found for N2 amplitudes (χ^2^(5) = 31.413, *p <* 0.001), and for N2 latencies (χ^2^(5) = 23.159, *p* < 0.001). Furthermore, statistically significant differences in the LoA between pairings were found for N2 amplitudes (χ^2^(5) = 19.222, *p* = 0.002), and for N2 latencies as well (χ^2^(5) = 14.841, *p* = 0.011). Post hoc analysis revealed several statistically significant differences in bias and LoA of N2 amplitude and latency estimations between pairings (see Fig 6 for details), but the error was generally smaller for pairings between the human observers and the DRIV algorithm. Furthermore, statistically significant differences in ICC between pairings were found for N2 amplitudes (χ^2^(5) = 27.730, *p <* 0.001) and N2 latencies (χ^2^(5) = 32.556, *p* < 0.001). Likewise, statistically significant differences in CV between pairings were found for N2 amplitudes (χ^2^(5) = 27.921, *p <* 0.001), and for N2 latencies as well (χ^2^(5) = 18.079, *p* = 0.003). Post hoc analysis revealed several statistically significant differences in ICC and CV of N2 amplitude and latency estimations between pairings (see Fig 6 and S4 Fig for details). Interestingly, the pairings between OBS1 and DRIV generally presented higher ICC and smaller CV values compared to the other possible pairings, including the pairing between the two human observers. As before, pairings with the WVLT algorithm usually resulted in in lower ICC and higher CV values.

#### P2 peak

**Fig 7:**
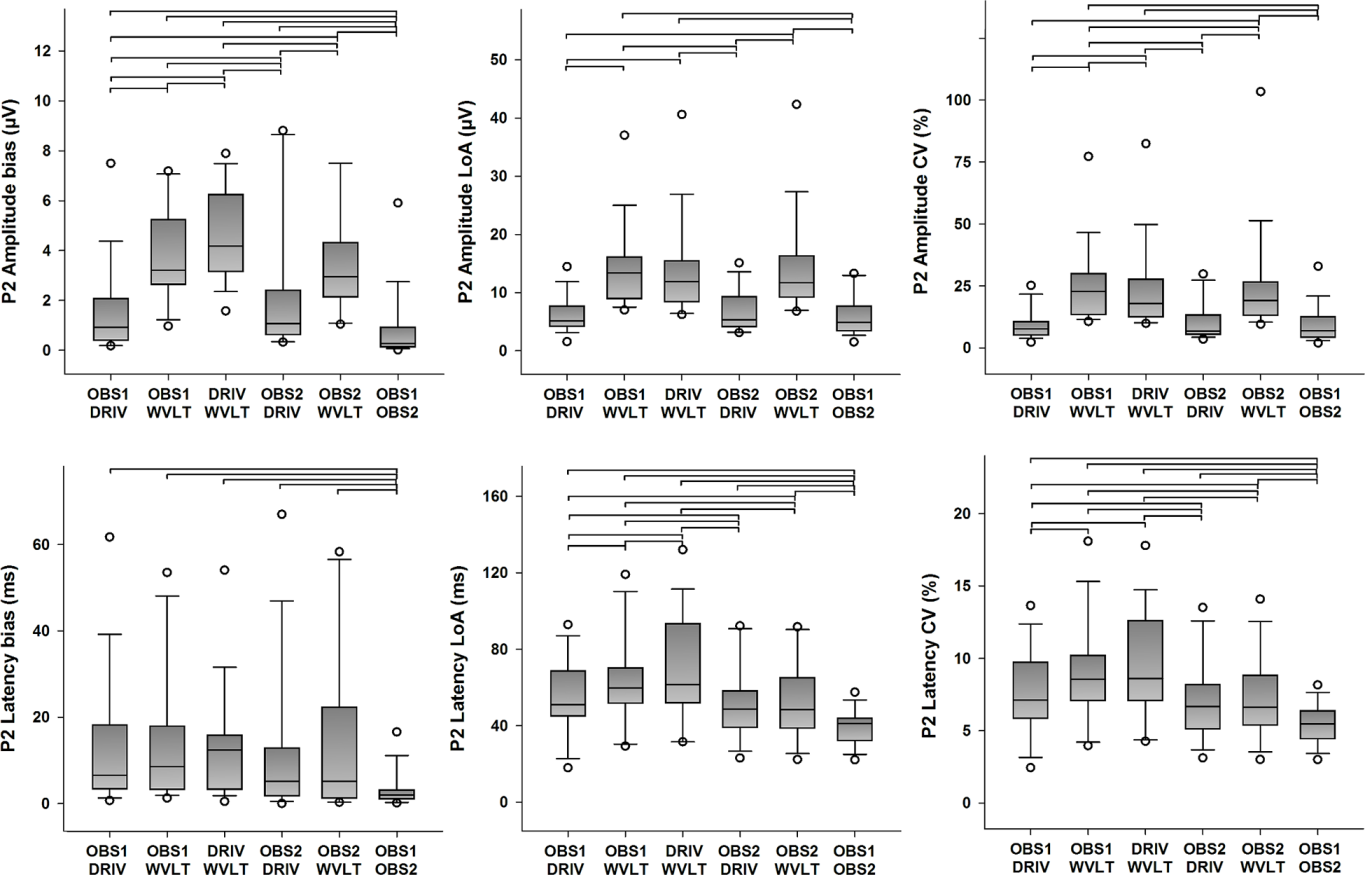
Quantitative agreement in the detection of the P2 peak (primary outcomes). The boundaries of the boxes indicate the 25^th^ and 75^th^ percentiles, the line within the box marks the median, the whiskers indicate the 10^th^ and 90^th^ percentiles and the circles above and below represent outliers (*n* = 16 for each index). Horizontal lines on top of the bars represent statistically significant post hoc differences between pairings (Student-Newman-Keuls, *p* < 0.05). LoA: limits of agreement, CV: coefficient of variation.

Descriptive statistics for the quantitative agreement from all possible pairings of detection methods of P2 amplitudes and latencies are presented in Fig 7 (primary outcomes) and S4 Fig (secondary outcome). Statistically significant differences in the systematic error between pairings were found for P2 amplitudes (χ^2^(5) = 52.393, *p <* 0.001), and for P2 latencies (χ^2^(5) = 15.464, *p* = 0.009). Furthermore, statistically significant differences in the random error between pairings were found for N1 amplitudes (χ^2^(5) = 54.821, *p <* 0.001), and for N1 latencies as well (χ^2^(5) = 42.143, *p <* 0.001). Post hoc analysis revealed several statistically significant differences in systematic and random error of P2 amplitude and latency estimations between pairings (see Fig 7 for details), but the error was generally smaller for pairings between the human observers and the DRIV algorithm. Overall, it was clear that the pairing between the human observers yielded overall smaller bias and LoA for the estimation of P2 amplitudes and latencies. Furthermore, statistically significant differences in ICC between pairings were found for P2 amplitudes (χ^2^(5) = 52.048, *p <* 0.001) and P2 latencies (χ^2^(5) = 48.115, *p* < 0.001). Likewise, statistically significant differences in CV between pairings were found for P2 amplitudes (χ^2^(5) = 60.429, *p <* 0.001), and for P2 latencies as well (χ^2^(5) = 42.143, *p* = 0.003). Post hoc analysis revealed several statistically significant differences in ICC and CV of P2 amplitude and latency estimations between pairings (see Fig 7 and S4 Fig for details). As for the N1 peak, the pairings between the human observers resulted in higher ICC and smaller CV values compared to the other possible pairings, followed by pairing between a human observer and the DRIV algorithm. Finally, pairings with the WVLT algorithm usually resulted in lower ICC and higher CV values.

### Effects of stimulation intensity on agreement

**Fig 8:**
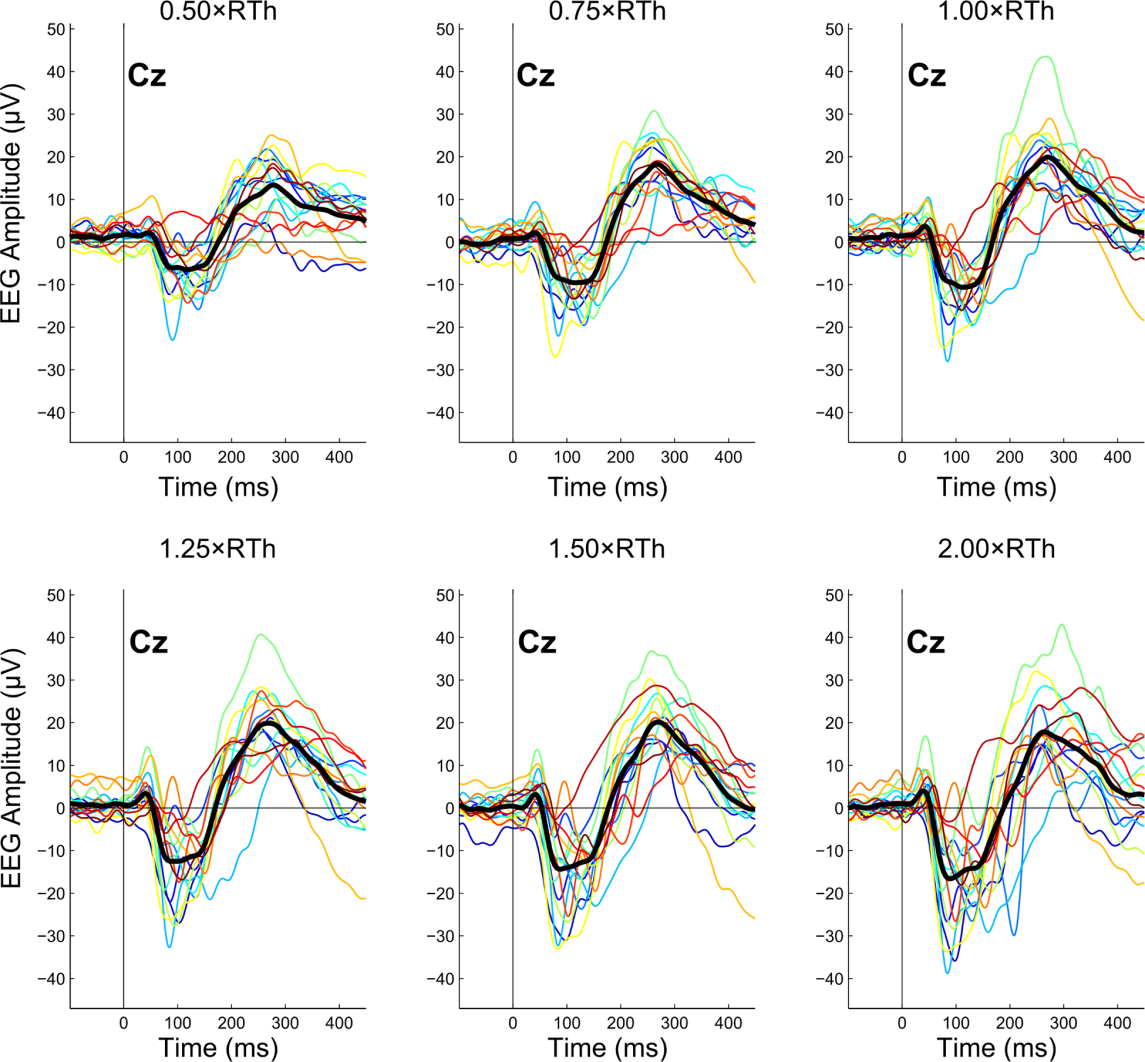
Effects of stimulation intensity on event-related potential (ERP) recordings. Each panel shows the average ERP of all available trials from each subject (color-coded) for a single stimulation intensity. The overlapping thick black line represents the grand average of all subjects (*n* = 16). RTh: nociceptive withdrawal reflex threshold.

Fig 8 shows the effect of stimulation intensity on the average ERP for each subject. In this regard, the best- and worst-performing pairings in terms of agreement were further selected to investigate the effects of stimulation intensity on categorical and quantitative agreement. From the previous analysis, these two pairing were OBS1-OB2 and DRIV-WVLT, respectively.

#### Effects of stimulation intensity on categorical agreement

There were no significant differences on categorical agreement (quantified as Cohen’s κ) due to stimulation intensity between OBS1 and OBS2 (N1: χ^2^(5) = 10.084, *p* = 0.073; N2: χ^2^(5) = 3.171, *p* = 0.674; P2: χ^2^(5) = 4.834, *p* = 0.436). Furthermore, there were also no significant differences on categorical agreement due to stimulation intensity between DRIV and WVLT either (N1, N2, P2: χ^2^(5) = 0, *p* = 1).

#### Effects of stimulation intensity on quantitative agreement

With regards to peak amplitudes, there were no significant differences on quantitative agreement (quantified as CV) due to stimulation intensity between OBS1 and OBS2 (N1: χ^2^(5) = 2.071, *p* = 0.839; N2: χ^2^(5) = 2.619, *p* = 0.758; P2: χ^2^(5) = 4.679, *p* = 0.456). Furthermore, there were no significant differences on quantitative agreement due to stimulation intensity between DRIV and WVLT either for the N2 and P2 peaks (N2: χ^2^(5) = 3.214, *p* = 0.667; P2: χ^2^(5) = 11.286, *p* = 0.046 but no significant post hoc comparisons). However, significant differences on quantitative agreement due to stimulation intensity between DRIV and WVLT were found for the N1 peak (χ^2^(5) = 16.736, *p* = 0.005). Post hoc analysis revealed that the CV was significantly lower (median CV: 14.7%) when the highest stimulation intensity (2.00×RTh) was used, compared to all other stimulation intensities, with median CVs ranging from 21.7% to 32.5% (*p* < 0.05).

In relation to peak latencies, there were no significant differences on quantitative agreement due to stimulation intensity between OBS1 and OBS2 (N1: χ^2^(5) = 4.000, *p* = 0.549; N2: χ^2^(5) = 2.714, *p* = 0.744; P2: χ^2^(5) = 7.612, *p* = 0.179). Furthermore, there were no significant differences on quantitative agreement due to stimulation intensity between DRIV and WVLT either for the N1 and N2 peaks (N1: χ^2^(5) = 7.110, *p* = 0.213; N2: χ^2^(5) = 9.357, *p* = 0.096). However, significant differences on quantitative agreement due to stimulation intensity between DRIV and WVLT were found for the P2 peak (χ^2^(5) = 20.607, *p <* 0.001). Post hoc analysis revealed that the CV was significantly higher (median CV: 9.1%) when the lowest stimulation intensity (0.50×RTh) was used, compared to all other stimulation intensities, with median CVs ranging from 6.6% to 7.4% (*p* < 0.05).

## Discussion

The aim of this study was to determine the categorical and quantitative agreement between manual and automated methods for single-trial detection and estimation of ERP features. To that end, sixteen healthy volunteers participated in a single experimental session, in which ERPs were elicited using electrical stimulation. ERPs were characterized by one or more peaks, named according to their latency: N1, N2 and P2. The presence/absence of an ERP peak (categorical outcome) and its amplitude and latency (quantitative outcome) in each single-trial recording were evaluated independently by two human observers and two automated algorithms. The results of this study demonstrated that the human observers generally presented the highest categorical and quantitative agreement, and that there were significantly large differences between detection and estimation of quantitative features among methods.

### Categorical agreement between manual and automated methods

To begin with, it is important to acknowledge that ERP responses are stochastic in nature, so there is no guarantee that the highest stimulation intensity will always evoke a response, or that the lowest stimulation intensity will never evoke a response, or that the intermediate stimulation intensity will evoke a response in 50% of the trials. Indeed, sometimes the lowest stimulation intensity will evoke a response and the highest intensity will not, and this will also change from subject to subject. Thus, the ‘true’ number of peaks in this experimental setting is unknown. That number could only be known for example in experiments involving simulated ERPs or in cases when there is a true gold standard to compare against, because in that case it could be precisely determined how many signals were include with and without true responses (and further, sensitivity and specificity values could be derived). In this study, four different assessment methods are presented where each of them reports a different number of peaks detected per subject, and the only evaluation that can be performed is related to how much these methods agree. Even if two or more given methods agree to a very high level on the number of existing peaks, it still cannot be stated with certainty that this number is close to the ‘true’ number of peaks, since these methods could very well be making the same mistakes during detection/estimation (i.e. the methods would agree on false positives or false negatives).

As expected, results showed that the median overall percent agreement ranged from very good to excellent in all cases (the median *p*_*o*_ is higher than 80% for all possible pairings of methods), particularly in the case of the P2 peak (where the median *p*_*o*_ is higher than 90%), since the binary choice (presence/absence) was easier to make when there was only one peak involved. When there was only one negative peak, there might have been a disagreement on whether it was N1 or N2, thus reducing the value of *p*_*o*_. In this regard, it has to be noted that the sample distribution for *p*_*o*_ presented a large variation from subject to subject, and that in some cases it could go below 20% for some subjects. Furthermore, there is an inherent problem with *p*_*o*_ as an agreement statistic: if the number of trials in which a peak is present is large relative to the number of trials in which a peak is absent. This situation is actually not uncommon in ERP studies, particularly those performed using stimulation intensities at or near detection threshold levels [32–34]. In these situations, the trials with a peak present will dominate the value of *p*_*o*_, giving a false impression of good performance [35]. In the extreme case, in which a method considers that a peak is present in all trials (as for example WVLT does when assessing P2), then *p*_*o*_ will be as high as the percentage of trials with a peak present as assessed by the second method, even though the two methods would disagree in all cases in which a peak was absent. This is clearly reflected in *p*_*pos*_ and *p*_*neg*_, which quantify the relative agreement when a peak is present and absent, respectively. It can readily be seen that whereas *p*_*o*_ was very similar to *p*_*pos*_, (i.e., when a peak is present), there were large differences in the assessment of the absence of a peak, as reflected by the low *p*_*neg*_ values (especially in the case of P2, with median values equal or very close to zero for pairings involving the WVLT algorithm).

Furthermore, neither of these indexes consider the agreement by chance, i.e., the level of agreement that would be expected if the assessments by the two methods were unrelated. For example, if two methods were deciding on the presence/absence of a peak at random, the expected agreement (*p*_*e*_) would still be 50% or even higher, if the methods tended to rate more towards either presence or absence of a peak. Indeed, large *p*_*e*_ values could be observed for all peaks and all possible pairings (and as before, particularly in P2). As a result, the κ statistic displayed values that are very low and even close to zero, particularly in the cases involving WVLT in the pairing, signaling that most of the agreement in those cases was just due to chance. Moreover, the median level of agreement for the pairings involving the two human observers was significantly higher than the level of almost all other possible pairings, with a median κ ranging from 0.4 to 0.7, indicating moderate to substantial agreement [35]. Additionally, the median κ between the DRIV algorithm and the human observers could be categorized as slight to fair, whereas all pairings involving the WVLT algorithm presented poor agreement [35].

### Quantitative agreement between manual and automated methods

In general, results showed that quantitative agreement (reflected as smaller values of bias and LoA) was also highest for pairings between the human observers, followed by pairings between the human observers and algorithms, and the lowest values for pairings between the two algorithms. In some cases, however, the agreement between the two human observers was not significantly higher than the agreement between one human observer and the DRIV algorithm. In absolute terms, the amplitude bias found in pairings between the human observers and the DRIV algorithm is practically negligible, with a median value below 1 μV in all cases. Pairings including the WVLT algorithm showed slightly larger amplitude bias with a median value around 5 μV, but with bias peaks about twice that number. In time, the median latency bias was usually lower than 10 ms, although the maximum values reached up to 60 ms in some cases (when there was at least one of the algorithms in the pairing), which likely indicates that the two methods in the pairing did not agree on whether the detected wave was N1 or N2. This also happened in the quantification of P2, probably because there were two maxima in the selected interval. Interestingly, this mismatch did not occur in the pairing between the two human observers, whose largest latency bias was less than 20 ms (significantly smaller than all other methods) but a final average latency difference close to zero (i.e. unbiased).

With regards to the LoA, the median and maximum values for amplitude were in most cases below 5 and 10 μV, respectively, for parings between the human observers and the DRIV algorithm, whereas for pairing involving the WVLT algorithm, the median and maximum LoA rose to 10 and 40 μV, respectively. In relation to latency, the median LoA were around 20 to 40 ms for N1 and N2 peaks (with maximum values around 60 to 80 ms) and 40 to 60 ms for the P2 peak (with maximum values around 80 to 120 ms). The other outcomes (ICC and CV) also displayed the same trend described before. In the majority of cases, median CV and ICC values for peak amplitudes and latencies were also highest for pairings between the human observers, although in some cases the difference was not significant when compared to a pairing between a human observer and the DRIV algorithm. In relation to the absolute differences, there are no reference limits or scales to define whether the absolute agreement is poor, fair, good or excellent (as Cohen’s κ has, for example); instead, it is usually left to the researcher’s own criteria to determine whether differences from a particular size are acceptable or not.

Nevertheless, a few studies have actually attempted to compare manual and automated assessment of single-trial ERP measurements before. Mayhew et al. compared the estimation of N2 and P2 peak amplitudes and latencies elicited by painful laser stimulation, performed by an automated algorithm based on multiple linear regression and a human observer, and reported very good to excellent correlation between the two approaches (*R* ^*2*^ ranging from 0.56 to 0.81) [10]. However, correlation as such is a measure of association, not agreement [28,36]. In time, Hatem et al. performed a similar analysis, but the evaluation was carried out using ICC [12]. They reported ICC values between 0.99 and 1 for two human observers, whereas the ICC values between manual and automated algorithm show a large disparity, covering the whole range of variation for ICC (0 to 1), although amplitudes in general displayed larger ICC values than latencies (as also noted in this study). Again, it has also been noted before that ICC is not a proper method for evaluating agreement [37], and that the evaluation of the performance of a method based on a comparison of the resulting ICC values with fixed, predefined thresholds may lead to inconsistent or erroneous results [27,38].

### Effects of stimulation intensity on agreement

The relationship between stimulation intensity and ERP features has been thoroughly explored in the past [39–42]. In general, ERP amplitude and latency correlate well with stimulation intensity, particularly at noxious levels [19,20]. However, the effects of stimulation intensity on agreement have not been explored before. The results from this study showed that the differences in agreement were in most cases not related to stimulation intensity. For categorical agreement in particular, this means that the number of peaks rated as present (or absent) at each stimulation intensity did not differ significantly between the human observers or between the algorithms. In the case of quantitative agreement, no significant differences due to stimulation intensity were found between the human observers for any of the peaks. However, some differences in quantitative agreement due to stimulation intensity were found between the two algorithms: N1 peak amplitudes presented less variation at the highest stimulation intensity, whereas P2 peak latencies showed more variation at the lowest stimulation intensity. While this behavior is consistent with earlier findings about the stability of the N1 and P2 peaks at those stimulation intensities, it also has to be noted that the absolute differences found are quantitatively small (7–18% in the case of N1 peak amplitudes and 2–3% for P2 peak latencies).

### Implications, advantages and limitation of the assessment methodologies

It is relevant to acknowledge that the fact that two particular methods have better agreement than any other two methods does not necessarily mean that the former perform a given task better than the latter. Indeed, other parameters, particularly validity and reliability, should also be taken into account in order to make such statement. The importance of agreement studies resides in the reference values they provide in relation to the maximum differences that can be expected if one method is applied instead of the other, which are not particularly obvious from the estimates usually reported in ERP studies (such as those presented in Table 1). This is especially relevant when there is a ‘gold standard’ methodology for assessment already established in the field. In particular, the opinion of an expert human observer can be considered as a first ‘gold standard’ [13,43]. In this regard, the categorical agreement for peak detection between two human observers (assessed with Cohen’s κ) ranges from moderate to substantial [35], and the present results are in line with the level of agreement between human observers found in similar studies from other areas [44]. It has to be noted, however, that the two human observers in this study work at the same research laboratory and were trained in a similar way, which is probably the most common situation when assessing agreement. In this regard, it can be hypothesized that experience plays an important role in agreement between human observers, and more experience would result in higher levels of agreement. Future studies could then try to determine if the level of agreement changes when observers with different experience level or trained in different laboratories are involved. Lastly, whereas the manual approach displays better agreement in the cases in which some of the trials do not present ERP responses, the obvious set-back compared to automated methods is that they are much more time- and resource-consuming, and it probably cannot be used when the number of trials is relatively large.

The level of agreement between the human observers was significantly higher than the one found between pairing involving automated algorithms, even in the case in which the algorithm (DRIV) purposely imitates the human decision-making process [21]. Naturally, not all algorithms follow this premise; in particular, the WVLT algorithm performs a pre-processing of the single-trial signal using wavelet filtering to reduce background noise and instead of directly measuring peak amplitudes and latencies from the resulting signal, the features are estimated using a multiple linear regression approach [11]. As mentioned before, this study did not attempt to establish which method is better, but how large can the difference between estimations be if a researcher chooses to use one algorithm instead of another, whichever these two algorithms might be. This is not at all an unlikely scenario, given the fact that many algorithms with this purpose are readily available online or can be requested from different laboratories, and many other are continuously being developed. So the question is how different the results would be for a particular study if one specific algorithm (or in more general terms, one specific method) for feature detection or estimation is used instead of another.

In this study, results showed that the differences in the estimated values were considerably large between all methods. These differences might be partially attributed to the complexity of the estimation task that for example can be seen when the levels of noise are so high that the random fluctuations in the signal are interpreted as peaks. However, they could also be explained by the intrinsic differences of each approach, e.g., the WVLT algorithm is not suited to detect cases in which one or several peaks are not present, and the EEG electrode configuration used in this study might not be optimal for the detection of overlapping peaks. In particular, the N2 peak elicited by electrical stimulation in the lower limb has been shown to be comprised by 2 subcomponents over temporal and fronto-central scalp areas [25], which might differentiate better from N1 if measured more contra-laterally. In this regard, the agreement between the humans observers and the WVLT algorithm (and any other algorithm in general) could readily be improved by including a set of rules to acknowledge the possibility of a peak being absent from a trial.

## Conclusion

In this study, typical values for the categorical agreement between manual and automated methods for detection/estimation of ERP features were presented, as well as reference values for the average and maximum differences that can be expected if one method is used instead of the others. The analysis of these values indicated that substantial care should be taken in the selection of the approach, since this choice may lead to considerably different results, and factors like stimulation intensity and particularly the expected number of trials with/without response can play a significant role in the size of these differences.

## Supporting Information

S1 Fig Categorical agreement in the detection of the N1 peak (secondary outcomes). The boundaries of the boxes indicate the 25^th^ and 75^th^ percentiles, the line within the box marks the median, the whiskers indicate the 10^th^ and 90^th^ percentiles and the circles above and below represent outliers (*n* = 16 for each index). Horizontal lines on top of the bars represent statistically significant post hoc differences between pairings (Student-Newman-Keuls, *p* < 0.05). *p*_*o*_: overall percent agreement, *p*_*e*_: chance percent agreement.
(TIF)

S2 Fig Categorical agreement in the detection of the N2 peak (secondary outcomes). The boundaries of the boxes indicate the 25^th^ and 75^th^ percentiles, the line within the box marks the median, the whiskers indicate the 10^th^ and 90^th^ percentiles and the circles above and below represent outliers (*n* = 16 for each index). Horizontal lines on top of the bars represent statistically significant post hoc differences between pairings (Student-Newman-Keuls, *p* < 0.05). *p*_*o*_: overall percent agreement, *p*_*e*_: chance percent agreement.
(TIF)

S3 Fig Categorical agreement in the detection of the P2 peak (secondary outcomes). The boundaries of the boxes indicate the 25^th^ and 75^th^ percentiles, the line within the box marks the median, the whiskers indicate the 10^th^ and 90^th^ percentiles and the circles above and below represent outliers (*n* = 16 for each index). Horizontal lines on top of the bars represent statistically significant post hoc differences between pairings (Student-Newman-Keuls, *p* < 0.05). *p*_*o*_: overall percent agreement, *p*_*e*_: chance percent agreement.
(TIF)

S4 Fig Quantitative agreement in the detection of the N1, N2 and P2 peaks. The boundaries of the boxes indicate the 25^th^ and 75^th^ percentiles, the line within the box marks the median, the whiskers indicate the 10^th^ and 90^th^ percentiles and the circles above and below represent outliers (*n* = 16 for each index). Horizontal lines on top of the bars represent statistically significant post hoc differences between pairings (Student-Newman-Keuls, *p* < 0.05). ICC: intraclass correlation coefficient.
(TIF)

S1 File Data derived from detection and estimation tasks. The compressed archive contains 4 different files in MATLAB *.mat format, named after each detection and estimation method. Within each file there are 16 structure arrays, each corresponding to a different subject. Each structure arrays has *j* rows and 10 columns, where *j* is the number of remaining trials after pre-processing. The columns represent respectively N1, N2 and P2 latency (in ms), N1, N2 and P2 amplitude (in μV), N1, N2 and P2 presence (where ‘1’ means a peak was detected and ‘0’ means a peak was not detected), and stimulation intensity (where ‘1’ to ‘6’ correspond to 0.50×RTh to 2.00×RTh in that order).
(ZIP)
