# Supplementary figures and images for: On the Agreement between Manual and Automated Methods for Single-Trial Detection and Estimation of Features from Event-Related Potentials
Source: PLoS One. 2015 Aug 10;10(8):e0134127. doi: 10.1371/journal.pone.0134127 (PMC4530886; doi:10.1371/journal.pone.0134127)

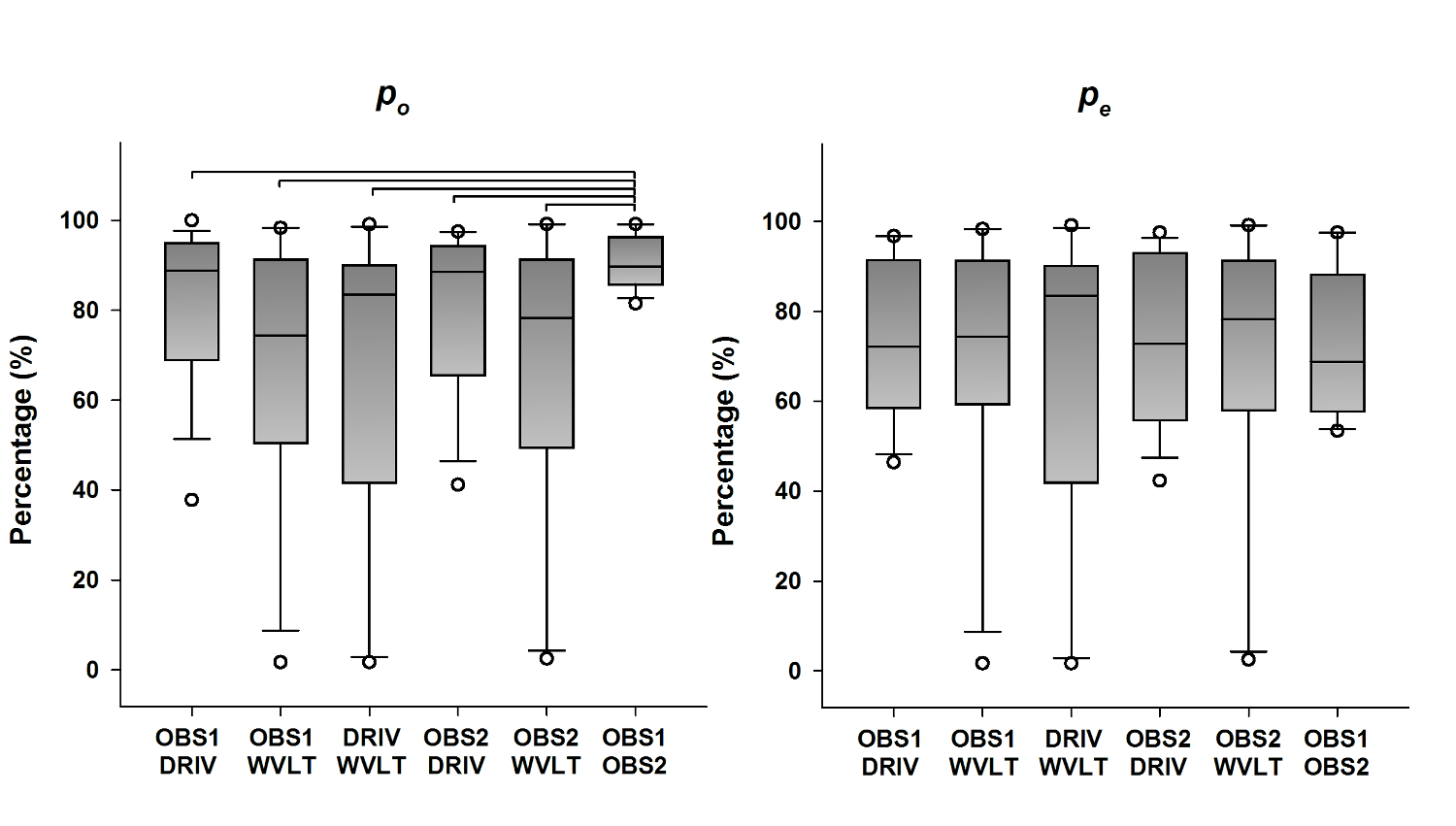

Supplement: S1 Fig — The boundaries of the boxes indicate the 25th and 75th percentiles, the line within the box marks the median, the whiskers indicate the 10th and 90th percentiles and the circles above and below represent outliers (n = 16 for each index). Horizontal lines on top of the bars represent statistically significant post hoc differences between pairings (Student-Newman-Keuls, p < 0.05). p o: overall percent agreement, p e: chance percent agreement. (TIF) [file pone.0134127.s001.TIF]

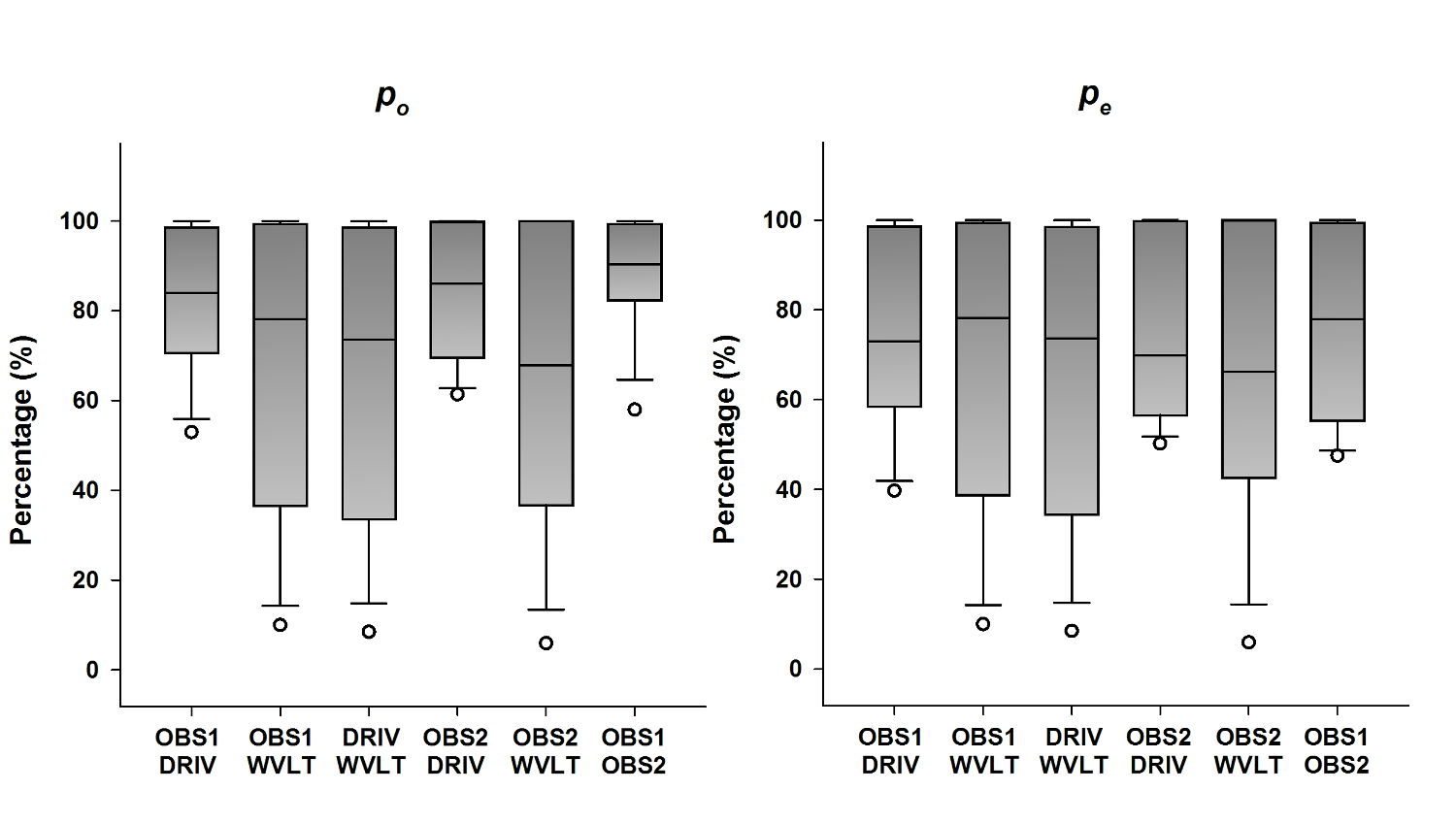

Supplement: S2 Fig — The boundaries of the boxes indicate the 25th and 75th percentiles, the line within the box marks the median, the whiskers indicate the 10th and 90th percentiles and the circles above and below represent outliers (n = 16 for each index). Horizontal lines on top of the bars represent statistically significant post hoc differences between pairings (Student-Newman-Keuls, p < 0.05). p o: overall percent agreement, p e: chance percent agreement. (TIF) [file pone.0134127.s002.TIF]

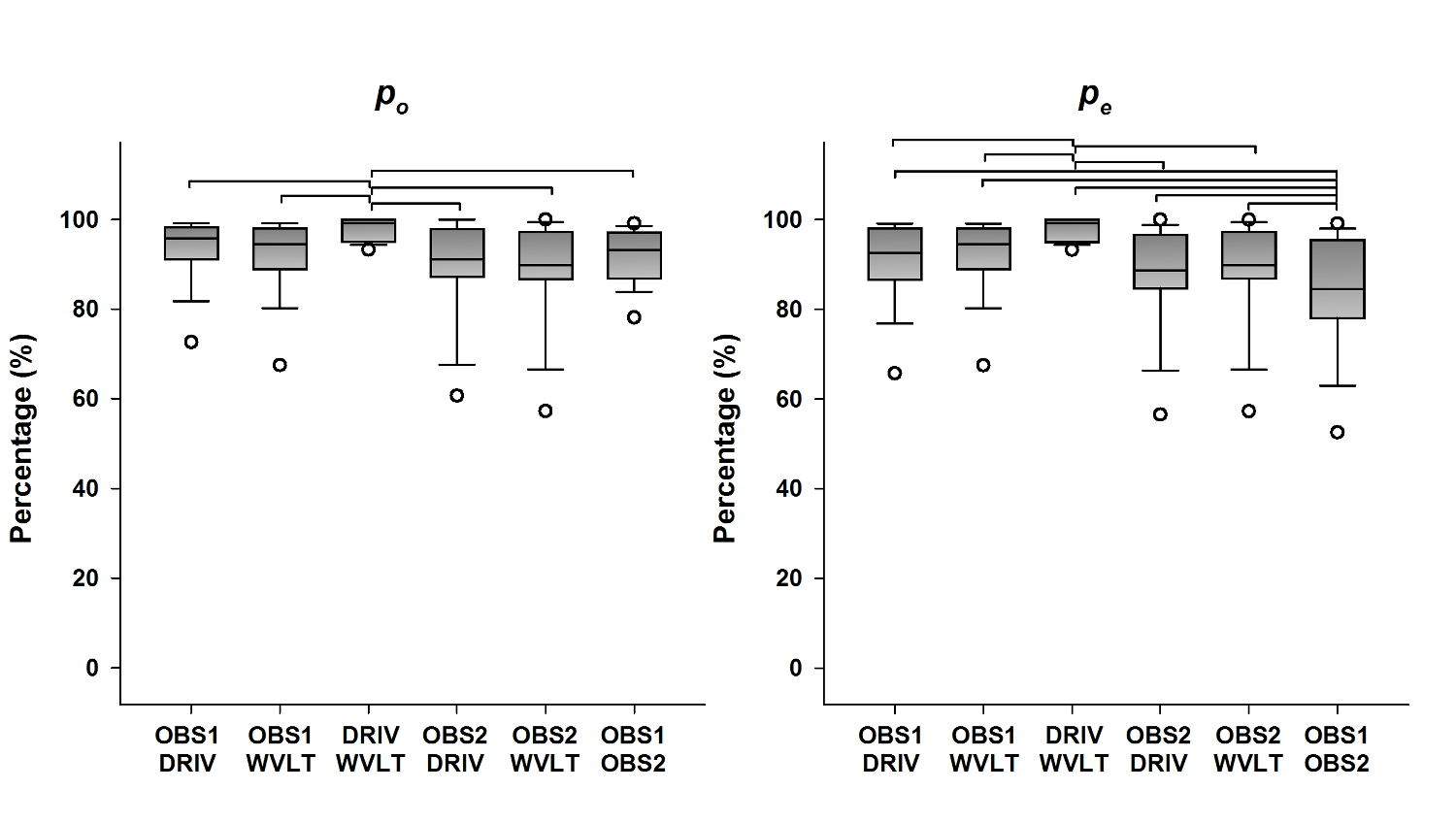

Supplement: S3 Fig — The boundaries of the boxes indicate the 25th and 75th percentiles, the line within the box marks the median, the whiskers indicate the 10th and 90th percentiles and the circles above and below represent outliers (n = 16 for each index). Horizontal lines on top of the bars represent statistically significant post hoc differences between pairings (Student-Newman-Keuls, p < 0.05). p o: overall percent agreement, p e: chance percent agreement. (TIF) [file pone.0134127.s003.TIF]

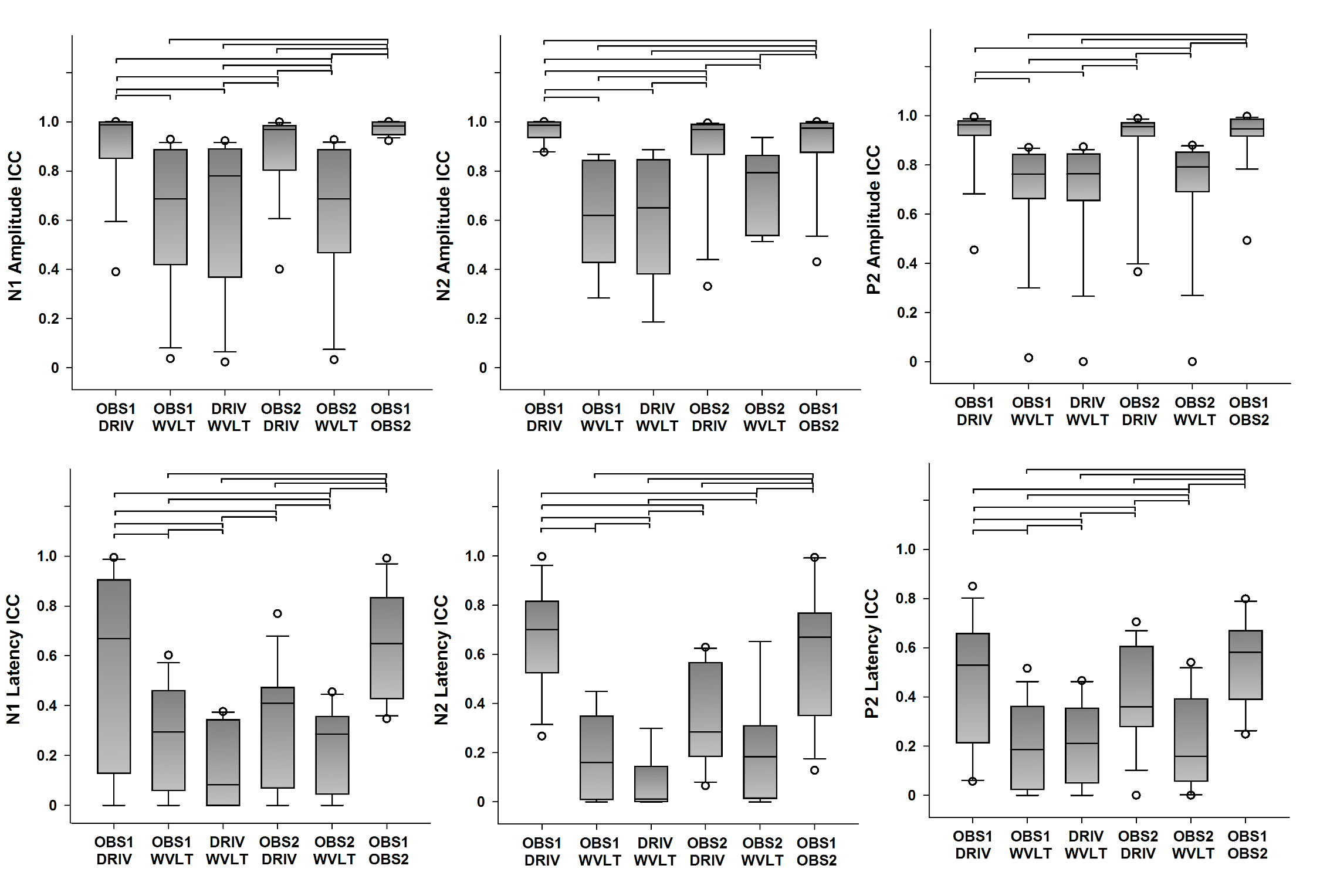

Supplement: S4 Fig — The boundaries of the boxes indicate the 25th and 75th percentiles, the line within the box marks the median, the whiskers indicate the 10th and 90th percentiles and the circles above and below represent outliers (n = 16 for each index). Horizontal lines on top of the bars represent statistically significant post hoc differences between pairings (Student-Newman-Keuls, p < 0.05). ICC: intraclass correlation coefficient. (TIF) [file pone.0134127.s004.TIF]
